# 24-month real-world persistence and compliance of denosumab versus alendronate among postmenopausal women with osteoporosis in Australia, Taiwan, South Korea, Hong Kong, and Singapore

**DOI:** 10.1007/s00774-026-01736-w

**Published:** 2026-06-17

**Authors:** Tang Ching Lau, Dong-Gune Chang, Chung-Hwan Chen, Shuang Huang, Edith M. C. Lau, Sheung-Wai Law, Young-Kyun Lee, Cae Tolman, Laura Canals, See-Hwee Yeo, Peter R. Ebeling

**Affiliations:** 1 Department of Medicine, Yong Loo Lin School of Medicine, National University of Singapore, Singapore, Singapore; 2 Department of Orthopedic Surgery, Inje University Sanggye Paik Hospital, College of Medicine, Inje University, Seoul, South Korea; 3 Department of Orthopedics and Orthopedic Research Center, Kaohsiung Medical University Hospital, College of Medicine, Regenerative Medicine and Cell Therapy Research Center, Kaohsiung Medical University, Kaohsiung, Taiwan; 4 Amgen Inc., Thousand Oaks, CA USA; 5 Hong Kong Orthopaedic and Osteoporosis Center for Treatment and Research, Hong Kong, China; 6 Department of Orthopaedics and Traumatology, The Chinese University of Hong Kong, Sha Tin, Hong Kong SAR, China; 7 Seoul National University Hospital, Seoul National University College of Medicine, Seoul, South Korea; 8 Amgen Australia, Sydney, Australia; 9 Department of Medicine, Amgen Europe, Amgen GmbH, Suurstoffi 22, 6343 Rotkreuz, Switzerland; 10 Real World Solutions, IQVIA Solutions Asia, Singapore, Singapore; 11 Department of Medicine, School of Clinical Sciences at Monash Health, Monash University, Melbourne, Australia

**Keywords:** Alendronate, Denosumab, Medication compliance, Medication persistence, Osteoporosis

## Abstract

**Introduction:**

To compare 24-month persistence and compliance of denosumab with alendronate in postmenopausal women with osteoporosis across 5 Asia–Pacific territories.

**Materials and methods:**

This prospective cohort study included women aged ≥ 50 years from Australia, Taiwan, South Korea, Hong Kong, and Singapore. Participants received either a bi-annual denosumab injection or weekly oral alendronate as osteoporosis treatment, based on physician judgment. Multivariable logistic regression assessed whether therapy (denosumab or alendronate) was significantly associated with treatment persistence and compliance over 24 months of follow-up. The models were adjusted for age, fracture history, baseline bone mineral density (BMD), prior osteoporosis therapy, and prior oral glucocorticoid treatment. Adjusted odds ratios (aORs) and 95% confidence intervals (CIs) were calculated.

**Results:**

Among 687 enrolled patients, 549 completed their 24-month visit (295 in denosumab and 254 in alendronate group). Patients in the denosumab group were older and a higher proportion previously received osteoporosis treatment. Overall treatment persistence and compliance were 74.9% and 56.6%, respectively. Patients prescribed with denosumab exhibited higher persistence (86.1% vs. 61.8%; *P* < 0.001) and compliance (62.4% vs. 50.0%; *P* = 0.004), compared with those on alendronate. Regression results also demonstrated that patients receiving denosumab were more likely to be persistent (aOR = 3.91; 95% CI 2.52–6.08), and compliant (aOR = 1.61; 95% CI 1.12–2.31). BMD T-scores improved in both the treatment groups after 24 months with no significant association between treatment type and change in T-score. No significant difference was also observed in the occurrence of adverse events between the two treatment cohorts. Patients on alendronate reported gastrointestinal issues more frequently, whereas those on denosumab had a higher incidence of bone and joint injuries.

**Conclusion:**

Postmenopausal women with osteoporosis were more likely to be persistent and compliant with a bi-annual denosumab injection, compared with weekly oral alendronate.

**Supplementary Information:**

The online version contains supplementary material available at https://doi.org/10.1007/s00774-026-01736-w.

## Introduction

Osteoporosis is a common bone disorder characterized by decreased bone density and deterioration of bone structure, leading to a heightened risk of fractures [1]. Based on data from 108 epidemiological studies, the global pooled estimate of osteoporosis prevalence stands at 19.7%, with over 9.0 million fractures occurring worldwide every year [2, 3]. Osteoporosis is prevalent among 30% of women (40 years of age or older) and 10% of men in middle- and high-income countries within the Asia–Pacific region [4]. In Australia, the prevalence of osteoporosis ranges from approximately 15–23% in women over the age of 50, while the estimates in South Korea are higher than 50% among women over the age of 60 [4]. The age-standardized hip fracture rates for women are notably high in Hong Kong, Singapore, and Taiwan, with incidence rates exceeding 300 per 100,000 individuals [5]. The number of hip fractures in Asia is estimated to rise from 1.1 million cases in 2018 to 2.6 million cases in 2050, accompanied by an increase in the economic burden from 9.5 billion to 15 billion United States dollars (USD) during the same period [6]. Hence, treatments that lower the risk of fractures play a crucial role in the management of osteoporosis. For optimal outcomes, patients need to persist on their osteoporosis therapies for the prescribed duration. However, achieving long-term medication compliance and persistence can be challenging [7].

Bisphosphonates such as alendronate are recommended as first-line treatment for osteoporosis [8]. A once-weekly 70 mg dose of oral alendronate is effective in increasing bone mineral density (BMD) and significantly lowers the risk of hip fractures if it is continued for several years [9]. However, poor drug absorption and gastrointestinal irritation associated with oral bisphosphonates may lead to lower persistence and compliance [10], thereby resulting in poorer outcomes [11]. Denosumab, administered as a 60 mg subcutaneous injection every 6 months (Q6M), is recommended as an alternative therapy for patients with a high risk of fractures and who are unable to tolerate oral therapy [12]. It is a human monoclonal antibody that acts by blocking the receptor activator of nuclear kappa-B ligand (RANKL), a key stimulator of osteoclast activity and a mediator of bone resorption [13]. Although both denosumab and bisphosphonates have demonstrated effectiveness in increasing BMD and reducing fracture risk [14], denosumab showed superiority in enhancing BMD at the hip, femoral neck, trochanter, lumbar spine, and one-third radius in postmenopausal women with osteoporosis over a 12-month period [15]. Furthermore, real-world evidence indicates that denosumab treatment resulted in a clinically meaningful reduction in fracture risk compared with alendronate (major osteoporotic, 39%; hip, 36%; non-vertebral, 43%; non-hip non vertebral, 50%; hospitalized vertebral, 30%) [16]. In addition, longer treatment with denosumab resulted in greater fracture reduction than alendronate. Furthermore, studies from Europe and the United States reported that patients on denosumab had higher treatment persistence and compliance, compared with those on oral bisphosphonates [17, 18]. This could be attributed to the absence of gastrointestinal side effects and patients' preference for parenteral administration [19].

Data on long-term medication persistence and compliance with denosumab or oral bisphosphonates is limited in the Asia–Pacific region, with only 2 studies from Taiwan published recently [20, 21]. In addition, treatment persistence and compliance may vary across geographies due to differences in healthcare access, drug reimbursement policies, clinical practice, and patient preferences [22]. Therefore, we conducted a two-year prospective, multicentre study between 2019–2023 to understand the persistence and compliance of denosumab injection Q6M versus oral alendronate weekly treatment among postmenopausal women in Australia, Taiwan, South Korea, Hong Kong, and Singapore. This study also aimed to investigate the incidence rate of spontaneously reported adverse events (AEs) and changes in BMD between the treatment groups at the 24-month timepoint. Our findings are expected to influence treatment decisions and improve outcomes in Asian patients with osteoporosis.

## Materials and methods

### Study design and study population

This multicentre, prospective cohort study was carried out across 33 sites in 5 Asia–Pacific territories, with the distribution of sites as follows: 11 in Australia, 6 in Taiwan, 5 in South Korea, 7 in Hong Kong, and 4 in Singapore. The enrolment period was from 25 June 2019 to 28 July 2021. Patients were recruited either from primary or specialty care settings, in accordance with territory-specific local practices. All eligible patients at each study site were invited in chronological order of their presentation for a routine clinical visit, and informed consent was collected for study participation. Participants were allocated to either the denosumab or alendronate cohort, depending on the medication prescribed for their osteoporosis treatment. The study had 3 timepoints of interest for data collection i.e., at enrolment, a 12-month visit, and a 24-month visit after enrolment. All follow-up visits adhered to the patient's routine standard of care. Data were recorded in electronic case report forms. A schematic representation of the study design is presented in Supplementary Fig. 1.

To be eligible for study inclusion, postmenopausal women must have obtained at least 1 prescription for denosumab or weekly oral alendronate before giving informed consent and must be enrolled no later than 4 weeks after the initial denosumab injection or first alendronate prescription for the ongoing treatment. The exclusion criteria included contraindications for using both denosumab and alendronate as specified in the approved local prescribing guidelines (e.g., hypocalcemia, hypersensitivity), participation in another research study involving any investigational procedure, device or medication in the last 6 months, as well as any condition that might impair the patient’s capacity to provide written informed consent.

Informed consent was obtained from all individual participants included in the study The study protocol received ethical approval from the Institutional Review Board of all local sites.

### Data collection

At enrolment, data on demographics, body mass index (BMI), smoking status, history of falls, history of fractures, current and previous osteoporosis treatment, BMD measurements, bone densitometer model, reimbursement status, and AEs were collected. BMD measurements and AEs (self-reported by patients) were also recorded at the 12-month and 24-month routine visits. Spontaneous AEs for both the denosumab and alendronate cohorts were classified using the Medical Dictionary for Regulatory Activities (MedDRA), version 22 [23].

### Estimation of 24-month treatment persistence and compliance

Patients were considered to be persistent with denosumab if they received the second, third, and fourth injections within 8 months or 240 days (6 months as scheduled and 2 months of allowed gap) from the previous injection. The formula used to calculate the gap period for denosumab injection was: ‘Denosumab_Gap_n_ = ‘Date of administration (n + 1^th^ injection of denosumab) – Date of administration (n^th^ injection of denosumab)’.

Patients were considered to be persistent with alendronate if there was no gap period of more than 60 days during the 24-month period (or 730 days) between the index date and the last dispensation date of alendronate, plus the days’ supply based on the quantity of pills for the last dispensation. The formula used to calculate the gap period based on alendronate dispensation dates and quantity supply was: ‘Alendronate_Gap_n_ = Date of dispensation (n + 1^th^ dispensation of alendronate)—Date of dispensation (n^th^ dispensation of alendronate)—7 days × Quantity of dispensation (number of 70 mg tablets)’.

Other studies that have been published on persistence with osteoporosis therapies have also used a permissible treatment gap period of 60 days (or 8 weeks) for denosumab and alendronate [17, 24]. Furthermore, in our study, switching treatment was regarded as a discontinuation of the therapy initially prescribed.

Participants were deemed to be compliant with denosumab treatment if they received all injections i.e., 4 injections within 24 months (or 730 days). Compliance at 24 months for weekly alendronate was measured using the medication possession ratio (MPR), which was calculated using the formula: ‘MPR = Total number of days on therapy (7 days multiplied by the quantity of dispensation)/Length of follow-up (730 days)’. Patients with an MPR ≥ 0.80 were regarded as compliant with treatment. This definition of compliance aligns with other comparable studies investigating compliance with osteoporosis treatment [17, 24].

### Statistical analysis

A target sample size of 77 patients per treatment group per territory would provide an 80% power (with type 1 error rate of 0.05) to detect a significant difference in our primary outcome of treatment persistence between denosumab and alendronate groups if a dropout rate of 10% and difference in persistence of 25% were assumed.

Baseline patient characteristics were summarized using descriptive statistics. Continuous variables were reported using mean, standard deviation (SD), median, and interquartile range (IQR), while categorical variables were presented using frequency and proportion. When comparing between groups, the chi-square test or Fisher’s exact test was used for categorical variables. For continuous variables, the Student’s t-test or Mann–Whitney U test was used.

The primary analysis for 24-month medication persistence and compliance was performed for patients across all territories and compared between treatment cohorts. Multivariable logistic regression was also used to examine the association between persistence (or compliance) and treatment prescribed. Based on clinical judgment and observed baseline characteristics that were significantly different between treatment groups, the regression models were adjusted for age, history of fracture, measurement of BMD, previous osteoporosis treatment, and prior treatment with glucocorticoids. Findings were presented using adjusted odds ratios (aORs) and 95% confidence intervals (CIs). Sensitivity analysis for treatment persistence was conducted by further adjusting for baseline drug reimbursement status when there were at least 10% subjects in reimbursement categories for both treatment groups. In addition, the gap period was varied by 30 or 90 days (instead of 60 days) to explore its impact on our results.

The change in BMD T-scores was measured both as an absolute change and as a percentage change from baseline. Multivariable linear regression was also conducted to examine the association between change in BMD T-scores and treatment groups. The regression models were adjusted for age, history of fracture, baseline BMD (at respective sites), prior treatment for osteoporosis, previous treatment with glucocorticoids, model of measuring device, history of falls, BMI, smoking status, and ethnicity. For each site (lumbar spine, total hip, femoral neck), findings were reported using regression coefficients and 95% CIs.

All statistical tests were 2-sided, and a *P*-value < 0.05 was considered statistically significant. Data analyses were performed using SAS software, version 9.4.

## Results

### Patient enrolment and baseline characteristics

Of the 705 patients screened, 687 were eligible and enrolled from 33 sites across 5 territories. A total of 549 (79.9%) patients subsequently completed their 24-month visit and were included in the final analysis. Among them, 295 patients were prescribed denosumab and 254 received alendronate. The territory-wise distribution of 549 patients was as follows: 131 from Hong Kong, 130 from South Korea, 124 from Australia, 101 from Taiwan, and 63 from Singapore (Supplementary Table 1 and Supplementary Fig. 2).

The mean age of all patients was 69.9 ± 8.8 years, ranging from 45–96 years (Table 1). Patients who received denosumab were older on average (70.8 ± 9.1 years) than those who received alendronate (68.9 ± 8.5 years; *P* = 0.005). The proportion of elderly patients ≥ 75 years was higher in the denosumab group (32.5%), compared with alendronate group (22.8%; *P* = 0.012). Most patients were of Chinese ethnicity (55.2%), followed by Korean (23.7%) and Caucasian (17.3%) ethnicities. The majority of patients (87.2%) did not receive prior oral glucocorticoid treatment. However, the proportion of those who received previous glucocorticoid therapy was higher in the denosumab cohort (18.0% vs. 6.3%; *P* < 0.001). Similarly, more than half of patients in the denosumab group (55.6%) received prior treatment for osteoporosis, compared with the alendronate group (37.0%; *P* < 0.001). In particular, a higher proportion of patients in the denosumab cohort had received parenteral bisphosphate therapy (26.8% vs. 14.9%; *P* = 0.027). Most patients (74.0%) in the study had baseline BMD assessments, with a higher percentage in the alendronate cohort (81.1%), compared with those prescribed denosumab (67.8%; *P* < 0.001). No notable differences were detected in other baseline demographic data or patient characteristics between the treatment groups. Characteristics such as BMI, smoking status, history of falls in the previous year, fracture history, parents’ history of fracture, initial BMD measurements, and drug reimbursement status were similar for both treatment cohorts.

**Table 1:** Demographics and clinical characteristics stratified by treatment group

Characteristic	Total (N = 549)		Denosumab (n = 295)		Alendronate (n = 254)		*P*-value^a^
	n	%	n	%	n	%	
Age (year)							
Mean (SD)	69.9 (8.8)		70.8 (9.1)		68.9 (8.5)		0.005*^b^
Min; Max	45.0; 96.0		45.0; 96.0		50.0; 93.0		
Median (IQR)	69.0 (64.0; 76.0)		70.0 (65.0; 77.0)		68.5 (63.0; 74.0)		
Age group, n (%)							
≥ 75 years old	154	28.1%	96	32.5%	58	22.8%	0.012*
< 75 years old	395	71.9%	199	67.5%	196	77.2%	
Ethnicity, n (%)							
Caucasian	95	17.3%	43	14.6%	52	20.5%	0.220^c^
Chinese	303	55.2%	167	56.6%	136	53.5%	
Korean	130	23.7%	71	24.1%	59	23.2%	
Malay	2	0.4%	2	0.7%	0	0.0%	
Indian	3	0.5%	3	1.0%	0	0.0%	
Others	16	2.9%	9	3.1%	7	2.8%	
Body mass index (kg/m^2^)							
Mean (SD)	23.8 (4.5)		23.7 (4.6)		23.9 (4.4)		0.507
Min; Max	14.6; 53.0		14.6; 44.6		15.8; 53.0		
Median (IQR)	23.1 (20.6; 26.0)		23.0 (20.4; 26.1)		23.1 (21.0; 26.0)		
Missing, n (%)	2 (0.4%)		1 (0.3%)		1 (0.4%)		
Smoking status, n (%)							
Current smoker	7	1.3%	2	0.7%	5	2.0%	0.455
Former smoker	35	6.4%	19	6.4%	16	6.3%	
Never smoker	506	92.2%	273	92.5%	233	91.7%	
Missing	1	0.2%	1	0.3%	0	0.0%	
Falls within last year, n (%)							
Yes	151	27.5%	73	24.7%	78	30.7%	0.125
One	113	74.8%	59	80.8%	54	69.2%	
Two to five	36	23.8%	14	19.2%	22	28.2%	
Five or more	2	1.3%	0	0.0%	2	2.6%	
No	397	72.3%	221	74.9%	176	69.3%	
Missing	1	0.2%	1	0.3%	0	0.0%	
Prior treatment with oral glucocorticoids, n (%)							
Yes	69	12.6%	53	18.0%	16	6.3%	< 0.001*
No	479	87.2%	241	81.7%	238	93.7%	
Missing	1	0.2%	1	0.3%	0	0.0%	
Prior treatment for osteoporosis, n (%)							
Yes^d^	258	47.0%	164	55.6%	94	37.0%	< 0.001*
Oral bisphosphonate therapy	150	58.1%	100	61.0%	50	53.2%	0.223
Calcitriol	12	4.7%	8	4.9%	4	4.3%	1.000^c^
Denosumab	60	23.3%	40	24.4%	20	21.3%	0.569
Injectable bisphosphonate therapy	58	22.5%	44	26.8%	14	14.9%	0.027*
Selective estrogen receptor modulators	29	11.2%	14	8.5%	15	16.0%	0.069
Calcitonin or elcatonin	5	1.9%	4	2.4%	1	1.1%	0.656^c^
Parathyroid hormone or teriparatide	23	8.9%	17	10.4%	6	6.4%	0.280
Strontium	12	4.7%	9	5.5%	3	3.2%	0.544^c^
Estrogen or hormone therapy	24	9.3%	9	5.5%	15	16.0%	0.005*
Other types^e^	16	6.2%	10	6.1%	6	6.4%	0.927
No	290	52.8%	130	44.1%	160	63.0%	
Missing	1	0.2%	1	0.3%	0	0.0%	
History of fracture, n (%)							
Yes	257	46.8%	133	45.1%	124	48.8%	0.402
Site of fracture^f^							
Hip	33	12.8%	17	12.8%	16	12.9%	0.977
Vertebral	131	51.0%	77	57.9%	54	43.5%	0.022*
Wrist	52	20.2%	25	18.8%	27	21.8%	0.553
Other sites	86	33.5%	42	31.6%	44	35.5%	0.507
Missing	2	0.8%	2	1.5%	0	0.0%	
Type of fracture^g^							
Osteoporotic	239	93.0%	121	91.0%	118	95.2%	0.189
Other types	29	11.3%	18	13.5%	11	8.9%	0.238
Missing	2	0.8%	2	1.5%	0	0.0%	
No	291	53.0%	161	54.6%	130	51.2%	
Missing	1	0.2%	1	0.3%	0	0.0%	
Parent history of fracture, n (%)							
Yes	70	12.8%	40	13.6%	30	11.8%	0.530
No	478	87.1%	254	86.1%	224	88.2%	
Missing	1	0.2%	1	0.3%	0	0.0%	
Measurement BMD							
Yes, n (%)	406	74.0%	200	67.8%	206	81.1%	< 0.001*
Model of measuring device, n (%)							
GE-lunar	185	45.6%	87	43.5%	98	47.6%	0.164
Hologic	168	41.4%	91	45.5%	77	37.4%	
Missing	53	13.1%	22	11.0%	31	15.0%	
Lumbar spine^h^							
T-score							
Mean (SD)	−2.3 (1.2)		−2.3 (1.3)		−2.4 (1.2)		0.827
Min; Max	−5.5; 3.5		−5.4; 2.6		−5.5; 3.5		
Median (IQR)	−2.5 (−3.1; −1.7)		−2.5 (−3.1; −1.7)		−2.5 (−3.1; −1.8)		
BMD value (g/cm^2^)							
Mean (SD)	0.9 (1.5)		1.0 (2.1)		0.8 (0.1)		0.995
Min; Max	0.5; 29.3		0.5; 29.3		0.5; 1.7		
Median (IQR)	0.8 (0.7; 0.9)		0.8 (0.7; 0.9)		0.8 (0.7; 0.9)		
Total hip^i^							
T-score							
Mean (SD)	−2.0 (0.8)		−2.1 (0.8)		−2.0 (0.8)		0.620
Min; Max	−5.5; 0.9		−4.3; 0.0		−5.5; 0.9		
Median (IQR)	−2.0 (−2.5; −1.5)		−2.0 (−2.6; −1.5)		−2.0 (−2.5; −1.4)		
BMD value (g/cm^2^)							
Mean (SD)	0.7 (0.1)		0.7 (0.1)		0.7 (0.1)		0.382
Min; Max	0.3; 1.1		0.3; 1.0		0.3; 1.1		
Median (IQR)	0.7 (0.6; 0.8)		0.7 (0.6; 0.8)		0.7 (0.7; 0.8)		
Femoral neck^j^							
T-score							
Mean (SD)	−2.4 (0.8)		−2.4 (0.8)		−2.4 (0.8)		0.507
Min; Max	−5.3; 0.2		−4.5; −0.1		−5.3; 0.2		
Median (IQR)	−2.5 (−2.9; −1.9)		−2.5 (−2.9; −2.0)		−2.4 (−2.8; −1.9)		
BMD value (g/cm^2^)							
Mean (SD)	0.6 (0.1)		0.6 (0.1)		0.6 (0.1)		0.381
Min; Max	0.2; 0.9		0.4; 0.9		0.2; 0.9		
Median (IQR)	0.6 (0.6; 0.7)		0.6 (0.6; 0.7)		0.6 (0.6; 0.7)		
No, n (%)	142	25.9%	94	31.9%	48	18.9%	
Missing, n (%)	1	0.2%	1	0.3%	0	0.0%	
Reimbursement status, n (%)							
Fully reimbursed	191	34.8%	110	37.3%	81	31.9%	0.358
Partially reimbursed	205	37.3%	104	35.3%	101	39.8%	
Self-funded	150	27.3%	78	26.4%	72	28.3%	
Missing	3	0.5%	3	1.0%	0	0.0%	

*BMD* bone mineral density, *IQR* interquartile range, *Max* maximum, *Min* minimum, *SD* standard deviation

^a^Unless otherwise specified, *P*-values were generated by the Mann–Whitney U test for continuous variables and the chi-square test for categorical variables

^b^The Student's t-test was used to compare the mean age of patients on denosumab and those on alendronate

^c^Fisher's exact test was used to compare patients on denosumab with those on alendronate

^d^A patient may have received multiple therapies. Hence, the sum of percentages for subcategories may exceed 100%

^e^Include records with an unknown name of therapy

^f^A patient may have multiple sites of fractures. Hence, the sum of percentages for subcategories may exceed 100%

^g^A patient may have multiple types of fracture. Hence, the sum of percentages for subcategories may exceed 100%

^h^The number of patients with a measurement of bone mineral density at the lumbar spine was 375, of whom 187 patients were on denosumab, and 188 patients were on alendronate

^i^The number of patients with a measurement of bone mineral density at the total hip was 372, of whom 182 patients were on denosumab, and 190 patients were on alendronate

^j^The number of patients with a measurement of bone mineral density at the femoral neck was 397, of whom 195 patients were on denosumab and 202 patients were on alendronate

*P < 0.05

### Overall 24-month treatment persistence and compliance

The overall persistence and compliance with osteoporosis treatment were 74.9% and 56.6%, respectively (Table 2). A significantly greater percentage of patients remained persistent (86.1% vs. 61.8%; P < 0.001) and compliant (62.4% vs. 50.0%; P = 0.004) with denosumab, compared with those receiving alendronate. These findings were largely consistent across territories. However, in South Korea, there was no significant difference in persistence between the treatment groups (P = 0.360), while the proportion of those compliant with alendronate was significantly higher (59.3% vs. 38.0%; P = 0.016). In addition, the proportions of those compliant in both treatment groups were comparable in Hong Kong (85.1% vs. 71.9%; P = 0.065).

**Table 2:** Osteoporosis treatment persistence and compliance at 24 months

	Total (N = 549)		Denosumab (n = 295)		Alendronate (n = 254)		*P*-value^c^
Persistence, n (%)^a^	n	%	n	%	n	%	
All territories	411	74.9%	254	86.1%	157	61.8%	< 0.001*
Australia	73	58.9%	50	84.7%	23	35.4%	< 0.001*
Taiwan	77	76.2%	49	87.5%	28	62.2%	0.003*
South Korea	101	77.7%	53	74.6%	48	81.4%	0.360
Hong Kong	109	83.2%	62	92.5%	47	73.4%	0.003*
Singapore	51	81.0%	40	95.2%	11	52.4%	< 0.001*^d^

	Total (N = 549)		Denosumab^e^ (n = 295)		Alendronate^f^ (n = 254)		*P*-value^c^
Compliance, n (%)^b^	n	%	n	%	n	%	
All territories	311	56.6%	184	62.4%	127	50.0%	0.004*
Mean (SD)	–		3.2 (1.2)		0.7 (0.3)		–
Min; Max	–		0.0; 4.0		0.0; 1.0		–
Median (IQR)	–		4.0 (1.0)		0.8 (0.6)		–
Australia	43	34.7%	29	49.2%	14	21.5%	0.001*
Mean (SD)	–		3.1 (1.2)		0.4 (0.3)		–
Min; Max	–		0.0; 4.0		0.0; 1.0		–
Median (IQR)	–		3.0 (1.0)		0.3 (0.5)		–
Taiwan	62	61.4%	40	71.4%	22	48.9%	0.021*
Mean (SD)	–		3.4 (1.2)		0.7 (0.3)		–
Min; Max	–		0.0; 4.0		0.0; 1.0		–
Median (IQR)	–		4.0 (1.0)		0.8 (0.5)		–
South Korea	62	47.7%	27	38.0%	35	59.3%	0.016*
Mean (SD)	–		2.6 (1.5)		0.8 (0.3)		–
Min; Max	–		0.0; 4.0		0.1; 1.0		–
Median (IQR)	–		3.0 (2.0)		0.9 (0.4)		–
Hong Kong	103	78.6%	57	85.1%	46	71.9%	0.065
Mean (SD)	–		3.6 (1.0)		0.8 (0.2)		–
Min; Max	–		0.0; 4.0		0.1; 1.0		–
Median (IQR)	–		4.0 (0.0)		0.9 (0.3)		–
Singapore	41	65.1%	31	73.8%	10	47.6%	0.040*
Mean (SD)	–		3.6 (0.7)		0.6 (0.4)		–
Min; Max	–		1.0; 4.0		0.0; 1.0		–
Median (IQR)	–		4.0 (1.0)		0.7 (0.6)		–

*IQR* interquartile range, *Max* maximum, *Min* minimum, *SD* standard deviation, – not applicable

^a^For the denosumab group, persistence at 24 months is defined as the patient receiving her 2nd, 3rd, and 4th injections within 8 months (6 months as scheduled and a 2-month allowed gap) of the previous injection. For alendronate, persistence at 24 months is defined as remaining on therapy with no gap > 60 days during the 24-month period between the index date and the last dispensation date of alendronate, plus the days’ supply based on the quantity of pills for the last dispensation

^b^For the denosumab group, compliance at 24 months is defined as having received 4 injections within 24 months. For alendronate, compliance at 24 months is defined as the medication possession ratio (total days on therapy divided by 730 days) ≥ 0.80

^c^Unless otherwise specified, *P*-values were generated by the chi-square test

^d^Fisher's exact test was used to compare persistence between patients on denosumab and those on alendronate

^e^Compliance is based on the number of injections received

^f^Compliance is based on the medication possession ratio

*P < 0.05

### Factors associated with treatment persistence

At 24 months, the treatment group, age, and history of fractures were significantly associated with treatment persistence (Table 3). Patients who received denosumab were almost 4 times more likely to be persistent with treatment, compared with those who received alendronate (aOR = 3.91; 95% CI 2.52–6.08, *P* < 0.001). Older age (aOR = 0.98; 95% CI 0.95–1.00, *P* = 0.043) and a history of fractures (aOR = 0.61; 95% CI 0.40–0.92, *P* = 0.019) were associated with lower likelihood of treatment persistence. Baseline BMD assessment, previous osteoporosis treatment, and past use of oral glucocorticoids were not associated with treatment persistence (*P* > 0.05).

**Table 3:** Adjusted odds ratios and 95% confidence intervals for osteoporosis treatment persistence at 24 months using multivariable logistic regression

Territory	Adjusted odds ratio^a^	95% CI	*P*-value	Reference group^b^
All territories (N = 549)				
Denosumab group	3.91	(2.52, 6.08)	< 0.001*	Alendronate
Age	0.98	(0.95, 1.00)	0.043*	–
History of fracture	0.61	(0.40, 0.92)	0.019*	No
Measurement of BMD	1.29	(0.80, 2.07)	0.297	No
Prior treatment for osteoporosis	1.44	(0.94, 2.20)	0.090	No
Prior treatment with oral glucocorticoids	0.98	(0.49, 1.97)	0.960	No
Australia (n = 124)				
Denosumab group	9.25	(3.72, 23.00)	< 0.001*	Alendronate
Age	0.99	(0.94, 1.05)	0.787	–
History of fracture	0.97	(0.42, 2.29)	0.953	No
Measurement of BMD	1.93	(0.50, 7.54)	0.343	No
Prior treatment for osteoporosis	1.38	(0.53, 3.58)	0.505	No
Prior treatment with oral glucocorticoids	0.82	(0.23, 2.90)	0.757	No
Taiwan (n = 101)				
Denosumab group	3.62	(1.27, 10.34)	0.016*	Alendronate
Age	0.99	(0.93, 1.05)	0.655	–
History of fracture	0.38	(0.11, 1.40)	0.146	No
Measurement of BMD	1.71	(0.47, 6.20)	0.416	No
Prior treatment for osteoporosis	1.00	(0.26, 3.79)	0.997	No
Prior treatment with oral glucocorticoids	1.88	(0.29, 12.00)	0.505	No
South Korea (n = 130)				
Denosumab group	1.13	(0.43, 2.99)	0.805	Alendronate
Age	0.94	(0.89, 1.00)	0.035*	–
History of fracture	0.39	(0.16, 1.00)	0.050*	No
Measurement of BMD	1.79	(0.56, 5.74)	0.327	No
Prior treatment for osteoporosis	2.38	(0.95, 6.01)	0.065	No
Prior treatment with oral glucocorticoids	0.82	(0.25, 2.67)	0.741	No
Hong Kong (n = 131)				
Denosumab group	3.63	(1.20, 10.97)	0.022*	Alendronate
Age	1.00	(0.95, 1.06)	0.924	–
History of fracture	1.14	(0.43, 3.03)	0.789	No
Measurement of BMD	2.60	(0.90, 7.56)	0.079	No
Prior treatment for osteoporosis	1.72	(0.55, 5.41)	0.354	No
Prior treatment with oral glucocorticoids	1.21	(0.05, 29.63)	0.909	No
Singapore (n = 63)				
Denosumab group	22.97	(3.73, 141.57)	< 0.001*	Alendronate
Age	0.95	(0.85, 1.06)	0.359	–
History of fracture	0.85	(0.19, 3.91)	0.834	No
Measurement of BMD	2.06	(0.37, 11.51)	0.412	No
Prior treatment for osteoporosis	0.41	(0.08, 2.06)	0.280	No
Prior treatment with oral glucocorticoids	4.34	(0.13, 151.08)	0.417	No

*BMD* bone mineral density, *CI* confidence interval, – not applicable

^a^Adjusted odds ratios were generated using multivariable logistic regression models. Treatment persistence was used as the dependent variable, while adjusting for treatment group, age, history of fracture, measurement of BMD, prior treatment for osteoporosis, and prior treatment with oral glucocorticoids

^b^The treatment groups were categorized as patients on denosumab and those on alendronate. The history of fracture includes those with fractures and those without fractures. Measurement of BMD includes those with BMD measurements and those without them. Patients who received prior treatment for osteoporosis includes individuals who were using and not using osteoporosis drugs before starting denosumab or alendronate treatment. Prior treatment with oral glucocorticoids includes individuals who were using or not using oral glucocorticoids before starting denosumab or alendronate treatment

*P < 0.05

When regression was performed for each territory, treatment with denosumab was significantly associated with persistence in territories including Australia (aOR = 9.25; 95% CI 3.72–23.00, *P* < 0.001), Taiwan (aOR = 3.62; 95% CI 1.27–10.34, *P* = 0.016), Hong Kong (aOR = 3.63; 95% CI 1.20–10.97, *P* = 0.022), and Singapore (aOR = 22.97; 95% CI 3.73–141.57, *P* < 0.001). In South Korea, only age (aOR = 0.94; 95% CI 0.89–1.00, *P* = 0.035) and history of fractures (aOR = 0.39; 95% CI 0.16–1.00, *P* = 0.050) were significantly associated with treatment persistence.

Overall results from the sensitivity analyses were largely in line with the primary analysis. Patients prescribed with denosumab were more likely to be persistent with treatment even after adjusting for reimbursement status (aOR = 3.92; 95% CI 2.52–6.10, *P* < 0.001) (Supplementary Table 2). When a gap period of 30 was used to estimate treatment persistence, patients in the denosumab group remained significantly more likely to be persistent (aOR = 5.27; 95% CI 3.45–8.05, *P* < 0.001) (Supplementary Table 3). This finding was similar when the gap period was increased to 90 days (aOR = 2.85; 95% CI 1.82–4.47, *P* < 0.001) (Supplementary Table 4).

### Factors associated with treatment compliance

Across all territories, the treatment group, history of fracture, previous osteoporosis treatment, and prior oral glucocorticoid use were significantly associated with treatment compliance at 24 months (Table 4). Patients who received denosumab were about 1.6 times more likely to be treatment compliant than those who received alendronate (aOR = 1.61; 95% CI 1.12–2.31, *P* = 0.010). Similarly, patients who received prior osteoporosis treatment were about 1.6-fold (aOR = 1.57; 95% CI 1.10–2.24, *P* = 0.013) more likely to be treatment compliant. However, patients with a fracture history (aOR = 0.63; 95% CI 0.45–0.90, *P* = 0.011), and those who received prior oral glucocorticoids (aOR = 0.55; 95% CI 0.32–0.93, *P* = 0.027) were less likely to be compliant with treatment. Age and baseline BMD measurements did not show significant associations with treatment compliance (*P* > 0.05).

**Table 4:** Adjusted odds ratios and 95% confidence intervals for osteoporosis treatment compliance at 24 months using multivariable logistic regression

Territory	Adjusted odds ratio^a^	95% CI	*P*-value	Reference group^b^
All territories (N = 549)				
Denosumab group	1.61	(1.12, 2.31)	0.010*	Alendronate
Age	1.00	(0.98, 1.02)	0.870	–
History of fracture	0.63	(0.45, 0.90)	0.011*	No
Measurement of BMD	0.96	(0.64, 1.44)	0.840	No
Prior treatment for osteoporosis	1.57	(1.10, 2.24)	0.013*	No
Prior treatment with oral glucocorticoids	0.55	(0.32, 0.93)	0.027*	No
Australia (n = 124)				
Denosumab group	3.03	(1.34, 6.86)	0.008*	Alendronate
Age	1.04	(0.99, 1.09)	0.154	–
History of fracture	0.73	(0.32, 1.64)	0.443	No
Measurement of BMD	1.38	(0.39, 4.89)	0.621	No
Prior treatment for osteoporosis	1.24	(0.52, 2.99)	0.627	No
Prior treatment with oral glucocorticoids	0.54	(0.17, 1.75)	0.306	No
Taiwan (n = 101)				
Denosumab group	2.43	(0.99, 5.97)	0.054	Alendronate
Age	0.99	(0.94, 1.05)	0.801	–
History of fracture	0.31	(0.10, 0.94)	0.038*	No
Measurement of BMD	1.49	(0.46, 4.81)	0.502	No
Prior treatment for osteoporosis	1.56	(0.47, 5.16)	0.466	No
Prior treatment with oral glucocorticoids	0.68	(0.18, 2.49)	0.555	No
South Korea (n = 130)				
Denosumab group	0.55	(0.25, 1.20)	0.131	Alendronate
Age	0.99	(0.94, 1.03)	0.573	–
History of fracture	0.58	(0.25, 1.31)	0.188	No
Measurement of BMD	1.78	(0.60, 5.25)	0.298	No
Prior treatment for osteoporosis	2.54	(1.14, 5.70)	0.023*	No
Prior treatment with oral glucocorticoids	0.60	(0.23, 1.60)	0.307	No
Hong Kong (n = 131)				
Denosumab group	1.82	(0.72, 4.59)	0.204	Alendronate
Age	1.01	(0.96, 1.05)	0.852	–
History of fracture	1.37	(0.57, 3.32)	0.485	No
Measurement of BMD	1.48	(0.56, 3.90)	0.433	No
Prior treatment for osteoporosis	1.15	(0.44, 3.00)	0.770	No
Prior treatment with oral glucocorticoids	4.40	(0.20, 96.59)	0.348	No
Singapore (n = 63)				
Denosumab group	2.71	(0.80, 9.18)	0.109	Alendronate
Age	1.05	(0.96, 1.14)	0.298	–
History of fracture	0.86	(0.26, 2.83)	0.798	No
Measurement of BMD	1.36	(0.41, 4.50)	0.611	No
Prior treatment for osteoporosis	1.15	(0.38, 3.53)	0.804	No
Prior treatment with oral glucocorticoids	0.39	(0.03, 5.59)	0.491	No

*BMD* bone mineral density, *CI* confidence interval, – not applicable

^a^Adjusted odds ratios were generated using multivariable logistic regression models. Treatment persistence was used as the dependent variable while adjusting for denosumab group, age, history of fracture, measurement of BMD, prior treatment for osteoporosis, and prior treatment with oral glucocorticoids

^b^The denosumab group was categorized as patients on denosumab and those on alendronate. The history of fracture includes those with fractures and those without fractures. Measurement of BMD includes those with BMD measurements and those without them. Prior treatment for osteoporosis includes individuals who were using and not using osteoporosis drugs before starting denosumab or alendronate treatment. Prior treatment with oral glucocorticoids includes individuals who were using or not using oral glucocorticoids before starting denosumab or alendronate treatment

*P < *0.05*

When regression was conducted for each territory, only patients prescribed denosumab in Australia had a significant likelihood of being compliant (aOR = 3.03; 95% CI 1.34–6.86, *P* = 0.008). Although the aORs showed a trend towards patients being more compliant with denosumab (compared with alendronate), the association between treatment group and compliance was not significant in Taiwan, Hong Kong and Singapore (*P* > 0.05). In South Korea, the only factor to have a significant association with treatment compliance was previous osteoporosis therapy (aOR = 2.54; 95% CI 1.14–5.70; *P* = 0.023).

### Changes in BMD T-score from baseline at 24-month visit

In the denosumab cohort, the mean baseline BMD T-scores at the lumbar spine, total hip, and femoral neck were −2.3 ± 1.1, −2.0 ± 0.7, and −2.3 ± 0.8, respectively (Table 5). After 24 months, T-scores at these sites improved to −1.9 ± 1.2, −1.8 ± 0.7, and −2.1 ± 0.7, respectively. The mean percentage change in T-score from baseline was the highest for the lumbar spine (25.8%), followed by the total hip (8.4%) and the femoral neck (3.8%). In the alendronate cohort, the respective mean baseline BMD T-scores at the lumbar spine, total hip, and femoral neck were −2.4 ± 1.1, −1.9 ± 0.8, and −2.2 ± 0.7, which were comparable to the baseline values in the denosumab cohort. After 24 months, T-scores at these sites improved to −2.0 ± 1.0, −1.8 ± 0.8, and −2.0 ± 0.8, respectively. The change from baseline was highest for the lumbar spine (21.8%), followed by the femoral neck (9.6%) and the total hip (8.2%).

**Table 5:** Changes in T-score at 24 months and their associations with osteoporosis treatment using multivariable linear regression

	Denosumab (n = 295)			Alendronate (n = 254)			Regression coefficient (95% CI)^a^
	Baseline mean (SD)	24-month mean (SD)	Change from baseline, mean (SD)	Baseline mean (SD)	24-month mean (SD)	Change from baseline, mean (SD)	
Lumbar spine							
T-score	−2.3 (1.1)	−1.9 (1.2)	0.4 (0.9)	−2.4 (1.1)	−2.0 (1.0)	0.4 (0.5)	−0.17 (−0.37, 0.04)
Percentage change in T-score from baseline (%)	–	–	25.8 (53.2)	–	–	21.8 (31.1)	−5.67 (−17.69, 6.35)
Total hip							
T-score	−2.0 (0.7)	−1.8 (0.7)	0.2 (0.2)	−1.9 (0.8)	−1.8 (0.8)	0.1 (0.2)	−0.03 (−0.09, 0.04)
Percentage change in T-score from baseline (%)	–	–	8.4 (13)	–	–	8.2 (18.9)	−0.99 (−5.93, 3.96)
Femoral neck							
T-score	−2.3 (0.8)	−2.1 (0.7)	0.2 (0.4)	−2.2 (0.7)	−2.0 (0.8)	0.2 (0.4)	0.02 (−0.09, 0.13)
Percentage change in T-score from baseline (%)	–	–	3.8 (27.9)	–	–	9.6 (20.2)	6.01 (−1.04, 13.06)

*BMD* bone mineral density, *CI* confidence interval, *SD* standard deviation, – not applicable

^a^Reference group for osteoporosis treatment comprised patients on alendronate. The linear regression model was adjusted for age, baseline BMD, history of fractures, prior treatment for osteoporosis, prior treatment with oral glucocorticoids, the model of the measuring device, history of falls within the last year, body mass index, smoking status, and ethnicity

*P < 0.05

Treatment type was not significantly associated with mean changes (Supplementary Table 5) and percentage changes (Supplementary Table 6) in T-scores from baseline for all sites. There was a consistent negative association between baseline BMD and absolute change in T-score (Supplementary Table 5). This means that it may be more likely for patients with lower baseline BMD to experience a greater improvement in T-score after treatment.

### Adverse events at 24-month visit

Overall, 142 (25.9%) patients reported AEs in the study (Table 6). AEs between the treatment cohorts were comparable, with 27.1% in the denosumab group and 24.4% in the alendronate group experiencing AEs (P = 0.470). Most AEs (76.4%) reported by patients were unrelated to the treatment. Only 5.2% (n = 7) of AEs reported in the denosumab cohort were related to or possibly related to treatment, compared with 30.6% (n = 38) of AEs in the alendronate cohort (P < 0.001). Treatment was withdrawn or interrupted due to 5.9% (n = 8) of AEs reported in the denosumab cohort, compared with 25.0% (n = 31) of AEs reported in the alendronate cohort (P < 0.001). There was no significant difference in outcomes between the treatment groups, with most of the patients recovering from the AEs (P = 0.078).

**Table 6:** Adverse events stratified by treatment group at 24 months

Type	Total (N = 259)		Denosumab (n = 135)		Alendronate (n = 124)		*P*-value^b^
	N	%	n	%	n	%	
Patient count, n (%)	549	100%	295	100%	254	100%	
No adverse event, n (%)	407	74.1%	215	72.9%	192	75.6%	0.470
With adverse event, n (%)	142	25.9%	80	27.1%	62	24.4%	
One	82	57.7%	52	65.0%	30	48.4%	
Two	29	20.4%	14	17.5%	15	24.2%	
Three or more	31	21.8%	14	17.5%	17	27.4%	
Relationship of AEs to treatment							
Not related	198	76.4%	123	91.1%	75	60.5%	< 0.001*
Related	25	9.7%	5	3.7%	20	16.1%	
Possibly related	20	7.7%	2	1.5%	18	14.5%	
Unlikely related	16	6.2%	5	3.7%	11	8.9%	
Action taken with study treatment							
No action taken	211	81.5%	120	88.9%	91	73.4%	< 0.001*^c^
Treatment withdrawn	26	10.0%	6	4.4%	20	16.1%	
Treatment interrupted	13	5.0%	2	1.5%	11	8.9%	
Not applicable	6	2.3%	4	3.0%	2	1.6%	
Change medication	1	0.4%	1	0.7%	0	0.0%	
Delayed	2	0.8%	2	1.5%	0	0.0%	
Outcome							
Recovered/Resolved	192	74.1%	104	77.0%	88	71.0%	0.078^c^
Not recovered/Not resolved	60	23.2%	25	18.5%	35	28.2%	
Fatal	6	2.3%	5	3.7%	1	0.8%	
Unknown	0	0.0%	0	0.0%	0	0.0%	
Recovered/Resolved with sequelae	1	0.4%	1	0.7%	0	0.0%	
Adverse events^a^, n (%)							
Gastrointestinal disorders	43	16.6%	10	7.4%	33	26.6%	< 0.001*
Infections and infestations	40	15.4%	25	18.5%	15	12.1%	0.153
Injury, poisoning and procedural complications^d^	34	13.1%	27	20.0%	7	5.6%	< 0.001*
Musculoskeletal and connective tissue disorders	34	13.1%	18	13.3%	16	12.9%	0.918
Nervous system disorders	19	7.3%	10	7.4%	9	7.3%	0.963
General disorders and administration site conditions	18	6.9%	7	5.2%	11	8.9%	0.244
Cardiovascular disorders	13	5.0%	6	4.4%	7	5.6%	0.658
Respiratory, thoracic, and mediastinal disorders	12	4.6%	4	3.0%	8	6.5%	0.182
Metabolism and nutrition disorders	11	4.2%	7	5.2%	4	3.2%	0.435
Skin and subcutaneous tissue disorders	8	3.1%	3	2.2%	5	4.0%	0.486^c^
Neoplasms benign, malignant, and unspecified (including cysts and polyps)	7	2.7%	3	2.2%	4	3.2%	0.713^c^
Ear and labyrinth disorders	5	1.9%	5	3.7%	0	0.0%	0.061^c^
Investigations	4	1.5%	3	2.2%	1	0.8%	0.623^c^
Eye disorders	3	1.2%	3	2.2%	0	0.0%	0.248^c^
Hepatobiliary disorders	2	0.8%	1	0.7%	1	0.8%	1.000^c^
Psychiatric disorders	2	0.8%	2	1.5%	0	0.0%	0.499^c^
Renal and urinary disorders	2	0.8%	1	0.7%	1	0.8%	1.000^c^
Blood and lymphatic system disorders	1	0.4%	0	0.0%	1	0.8%	0.479^c^
Missing	1	0.4%	0	0.0%	1	0.8%	0.479^c^
Serious adverse events, n (%)							
Hospitalization	81	31.3%	49	36.3%	32	25.8%	0.069
Gastrointestinal disorders	5	6.2%	2	4.1%	3	9.4%	0.379^c^
Infections and infestations	17	21.0%	10	20.4%	7	21.9%	0.874
Injury, poisoning and procedural complications^d^	16	19.8%	14	28.6%	2	6.3%	0.014*
Musculoskeletal and connective tissue disorders	5	6.2%	5	10.2%	0	0.0%	0.151^c^
Nervous system disorders	6	7.4%	3	6.1%	3	9.4%	0.676^c^
General disorders and administration site conditions	4	4.9%	0	0.0%	4	12.5%	0.022*^c^
Cardiovascular disorders^d^	8	9.9%	3	6.1%	5	15.6%	0.253^c^
Respiratory, thoracic, and mediastinal disorders	4	4.9%	2	4.1%	2	6.3%	0.646^c^
Metabolism and nutrition disorders	6	7.4%	5	10.2%	1	3.1%	0.395^c^
Neoplasms benign, malignant, and unspecified (including cysts and polyps)	5	6.2%	2	4.1%	3	9.4%	0.379^c^
Ear and labyrinth disorders	2	2.5%	2	4.1%	0	0.0%	0.516^c^
Investigations	1	1.2%	0	0.0%	1	3.1%	0.395^c^
Hepatobiliary disorders	1	1.2%	1	2.0%	0	0.0%	1.000^c^
Missing	1	1.2%	0	0.0%	1	3.1%	0.395^c^
Death	0	0.0%	0	0.0%	0	0.0%	-
Other medically important event^e^	2	0.8%	2	1.5%	0	0.0%	0.499^c^
Life-threatening	0	0.0%	0	0.0%	0	0.0%	-
Significant disability	0	0.0%	0	0.0%	0	0.0%	-
Congenital anomaly or birth defect	0	0.0%	0	0.0%	0	0.0%	-

– Not applicable

^a^Adverse events were grouped based on their System Organ Classes derived from the Medical Dictionary for Regulatory Activities Version 22

^b^Unless otherwise specified, *P*-values were generated by the chi-square test for categorical variables

^c^Fisher's exact test was used to determine the association between variables related to adverse events and treatment groups

^d^Include adverse events related to injuries (e.g. fractures or lacerations), poisonings (e.g. overdoses or toxicities), and complications that arise because of medical procedures (e.g. surgical complications or post-operative infections)

^e^Includes neoplasms

*P < 0.05

Most AEs were not considered to be serious. Gastrointestinal disorders were more common in the alendronate cohort than in the denosumab cohort (n = 33, 26.6% vs. n = 10, 7.4%; *P* < 0.001). Conversely, AEs classified as 'injury, poisoning, and procedural complications' occurred more frequently in the denosumab cohort (n = 27, 20.0% vs. n = 7, 5.6%; *P* < 0.001). AEs in this category include injuries (such as fractures or cuts), poisonings (such as overdoses or toxicities), and complications from medical procedures (such as surgical complications or infections after surgery). The incidence of other AEs was comparable between the 2 cohorts.

The incidence of serious adverse events resulting in hospitalization was similar between the treatment cohorts (36.3% in denosumab group vs. 25.8% in alendronate group; *P* = 0.069). No deaths or life-threatening incidents were reported in this study.

## Discussion

This multicentre, prospective cohort study is the first to examine the 24-month treatment persistence and compliance of denosumab injection Q6M versus weekly oral alendronate in postmenopausal women with osteoporosis across 5 territories in the Asia–Pacific region. The patients prescribed denosumab had better persistence and compliance than those treated with alendronate. Multivariable regression models also showed that medication persistence and compliance were significantly associated with denosumab treatment across all territories, after accounting for potential confounders. Findings from sensitivity analyses which varied the gap period for estimation of persistence were generally in line with our primary analysis. Even though improvement in BMD T-scores was observed in both treatment groups after 24 months, there was no significant association detected between type of treatment and change in T-score at the lumbar spine, total hip or femoral neck. In addition, there was no significant difference in the occurrence of AEs between the denosumab and alendronate cohorts.

The baseline demographics and clinical characteristic were generally comparable between patients in the denosumab and alendronate groups. However, patients treated with denosumab were significantly older and more likely to have received prior osteoporosis treatment than those treated with alendronate. These findings align with another real-world, population-based cohort study in Canada [25]. The increased likelihood of previous osteoporosis treatment in the denosumab cohort may be explained by the diminishing efficacy of bisphosphonates in raising BMD over long treatment periods, which leads physicians to prescribe alternative therapies such as denosumab to sustain or improve BMD in patients with osteoporosis [26]. Another possible reason could be adherence to local osteoporosis treatment guidelines and reimbursement policies, such as those from the Osteoporosis Society of Hong Kong, which recommend denosumab as an alternative for postmenopausal patients who cannot use bisphosphonates because of contraindications, tolerability issues, or inadequate clinical responses [27].

Our study results showed that patients prescribed denosumab were more persistent and compliant with treatment, compared with alendronate group. Denosumab is administered Q6M, hence it is potentially more convenient in utilization than alendronate, which is taken weekly or daily [12]. The high frequency of gastrointestinal side effects and specific requirements for the administration of oral bisphosphonates, such as swallowing the pills on an empty stomach with water (preferably 30 min prior to eating) and staying in an upright posture for a minimum of 30 min [28], have been identified as major factors for the discontinuation of bisphosphonates [29]. The use of denosumab avoids these issues and its longer dosing schedule may help promote treatment persistence and compliance over the long-term. Clinical practice guidelines suggest that patients who may face difficulties being persistent and compliant with oral bisphosphonates could consider denosumab as a potential treatment option [30].

The persistence and compliance rates among patients receiving denosumab in our study are consistent with previous published studies [24, 31]. We observed an 86.1% persistence rate and 62.4% compliance rate for denosumab over 24 months, which are comparable to the results reported by a European study (persistence: 75.1–86.0%; compliance: 62.9–70.1%) [19]. However, our persistence rate was higher, compared with those reported by a French prospective cohort study (72%) [32], and a population-based cohort study in Denmark (71.9%) [33]. A retrospective study from Austria also reported long-term (over 3 years) real-world treatment persistence with denosumab at over 70% [34]. On the other hand, the persistence (61.8%) and compliance (50.0%) with alendronate, as estimated in our study, were comparatively lower than earlier research conducted in Asia [35, 36]. For example, a study conducted in Singapore reported 24-month persistence and compliance for alendronate and risedronate at 69% and 72%, respectively [35]. Similarly, another Korean study estimated 24-month persistence and compliance with oral bisphosphonates to be 63.3% and 60.1%, respectively [36]. Notably, both of these studies were conducted before the COVID-19 pandemic. As our present study was performed during the pandemic, it is possible that treatment for less life-threatening chronic illnesses like osteoporosis was postponed or delayed due to strict lockdown measures or patients’ fear of infection when visiting healthcare facilities [37]. This could have a larger negative impact on persistence or compliance with medications which require more frequent collection and administration. Although guidance on osteoporosis management suggests that there is no evidence of a direct interaction between COVID-19 vaccination and denosumab or oral bisphosphonates, a gap of 4–7 days is recommended between COVID-19 vaccination and denosumab injection [38]. It is also plausible that differences in treatment persistence or compliance reported in various studies and across territories may stem from how they are defined and calculated, duration of follow-up, as well as characteristics of the study population.

Interestingly, our study findings did not show significantly higher treatment persistence and compliance in Korean patients who received denosumab, compared with those prescribed alendronate. This is contrary to prior reports from various geographies demonstrating higher treatment persistence with denosumab compared to alendronate [17, 39]. One explanation could be that in South Korea, the National Health Insurance criteria for reimbursement of osteoporosis therapy is restricted to those patients with BMD T-score ≤ −2.5 [40]. Thus, patients who are prescribed denosumab and improve their BMD T-score to between −2.0 and −2.5 are no longer covered by insurance and are likely to either switch or discontinue their treatment [41].

Although our study indicated that patients treated with denosumab had better persistence and compliance than those treated with alendronate, no significant improvement in BMD T-score was observed across various skeletal sites, including the lumbar spine, total hip, and femoral neck. A prior meta-analysis of 10 randomized controlled trials (RCTs) among patients with osteoporosis reported that denosumab improved BMD significantly at the total hip, lumbar spine, femoral neck, and trochanter, compared with alendronate after 24 months [42]. In addition, a pooled analysis of 4 RCTs among postmenopausal women who previously received oral bisphosphonates reported that denosumab significantly improved BMD at the lumbar spine, total hip, femoral neck, and 1/3 radius over 12 months, compared with other bisphosphonates including oral alendronate, risedronate, ibandronate, and intravenous zoledronic acid [43]. There are a few possible reasons why we did not detect a significant improvement in BMD T-score among patients prescribed denosumab, compared with those in the alendronate group. Firstly, as we performed our sample size calculation based on the primary outcome of medication persistence, the study was not statistically powered to detect a difference in T-scores. Secondly, in our regression models, T-scores from the alendronate group were used as reference to compare against measurements from the denosumab group. As both alendronate and denosumab are known to improve BMD, it may be more challenging for denosumab to demonstrate its superiority over alendronate in the real world, even with better treatment persistence or compliance. Lastly, our results were likely affected by missing data. About 32% and 19% of patients did not have baseline BMD measurements in the denosumab and alendronate cohorts, respectively.

The majority of patients in the study did not experience AEs over a 24-month follow-up period. Gastrointestinal disorders were more common in the alendronate cohort than in the denosumab cohort. Conversely, bone and joint injuries such as fractures and dislocations, as well as procedural-related complications (all categorized under injury, poisoning, and procedural complications in MedDRA) were more frequently reported by patients receiving denosumab. Gastrointestinal side effects may contribute towards the higher incidences of treatment interruption and withdrawal in the alendronate cohort [44]. The higher frequency of bone and joint injuries in the denosumab group could be due to the significantly higher proportion of elderly patients (compared with alendronate group), while procedural-related complications may arise from the subcutaneous administration required for denosumab. It is plausible that these injuries could lead to more hospitalizations. The reported AEs may also be influenced by other comorbidities present in our study population, such as coronary heart disease or arthritis which is commonly found in adults with osteoporosis [45]. However, as this study did not collect data on comorbid conditions, it is uncertain if these were balanced between the treatment cohorts.

Our findings should be interpreted with caution due to several limitations. Firstly, the study duration between 2019–2021 coincided with the COVID-19 pandemic, which resulted in widespread lockdowns, travel restrictions, and social distancing measures [46]. These measures have affected healthcare access and disrupted routine medical appointments [46], thus impacting patient enrolment and follow-up. For example, only 69 patients were enrolled into the 2 treatment groups in Singapore (instead of the target number of 154 patients). Although the study was designed to reduce missing data by minimizing the number of required visits and collecting only the essential information at each visit [47], our study still included a significant proportion of patients (26% in total) without baseline BMD measurements. Secondly, the study results may have been influenced by the small sample sizes within each territory. To address this limitation, pooled data from all territories were included in the analysis. Thirdly, high loss to follow-up were observed, particularly in Taiwan and Australia, which posed difficulties in maintaining an adequate number of participants for analysis and also limited the feasibility of conducting further subgroup analyses. Fourthly, the observed treatment persistence and compliance may be affected by how they are defined and calculated. At present, there is no standardized definition or methodology for estimating medication persistence and adherence [48]. It is also unclear whether patients prescribed alendronate actually picked up the medication or adhered to the medication regimen at home. Finally, our study findings may be influenced by differences in BMD measuring device across the study centers [49]. Despite adjustments for potential confounders, residual confounding could still affect our regression results.

## Conclusion

This study showed that postmenopausal women with osteoporosis were more likely to be persistent and compliant with denosumab than with weekly oral alendronate after 24 months. Both treatments improved BMD T-scores, but no significant association was found between the type of treatment and T-score changes at the lumbar spine, total hip, or femoral neck. Most patients did not report AEs from the therapies. The findings strengthen our understanding of medication persistence and compliance among postmenopausal women with osteoporosis in the Asia–Pacific region, as well as provide useful insights to guide treatment choices, particularly for patients at high risk of non-adherence or non-compliance with oral bisphosphonates.

## Supplementary Information

Below is the link to the electronic supplementary material.

Supplementary file1 (DOCX 120 kb)

## Data Availability

Qualified researchers may request data from Amgen clinical studies. Complete details are available at the following: https://wwwext.amgen.com/science/clinical-trials/clinical-data-transparency-practices/clinical-trial-data-sharing-request/
