# Supplementary material for: 24-month real-world persistence and compliance of denosumab versus alendronate among postmenopausal women with osteoporosis in Australia, Taiwan, South Korea, Hong Kong, and Singapore
Source: J Bone Miner Metab. 2026 Jun 17;44(5):610–27. doi: 10.1007/s00774-026-01736-w (PMC13723669; doi:10.1007/s00774-026-01736-w)
Supplement: Supplementary file 1 [file 774_2026_1736_MOESM1_ESM.docx]

**Supplementary Information**

**24-Month Real-World Persistence and Compliance: Denosumab Versus Alendronate in Postmenopausal Women With Osteoporosis**

Tang Ching Lau^1^, Dong-Gune Chang^2^, Chung-Hwan Chen^3^, Shuang Huang^4^, Edith MC Lau^5^, Sheung-Wai Law^6^, Young-Kyun Lee^7^, Cae Tolman^8^, Laura Canals^9^, See-Hwee Yeo^10^, Peter R. Ebeling^11^

^1^ Department of Medicine, Yong Loo Lin School of Medicine, National University of Singapore, Singapore, Singapore

^2^ Department of Orthopedic Surgery, Inje University Sanggye Paik Hospital, College of Medicine, Inje University, Seoul, South Korea

^3^ Department of Orthopedics and Orthopedic Research Center, Kaohsiung Municipal Ta-Tung Hospital and Kaohsiung Medical University Hospital, College of Medicine; Regeneration Medicine and Cell Therapy Research Center, Kaohsiung Medical University, Kaohsiung, Taiwan

^4^ Amgen Inc., Thousand Oaks, California, United States of America

^5^ Hong Kong Orthopaedic and Osteoporosis Center for Treatment and Research, Hong Kong

^6^ Department of Orthopaedics and Traumatology, The Chinese University of Hong Kong, Hong Kong SAR, China

^7^ Seoul National University Hospital, Seoul National University College of Medicine, Seoul, South Korea

^8^ Amgen Australia, Sydney, Australia

^9^ Department of Medicine, Amgen Europe, Switzerland

^10^ Real World Solutions, IQVIA Solutions Asia, Singapore, Singapore

^11^ Department of Medicine, School of Clinical Sciences at Monash Health, Monash University, Melbourne, Australia

**Correspondence:**

Laura Canals

Address: Amgen GmbH, Suurstoffi 22, 6343 Rotkreuz, Switzerland

Telephone: +41 791539645

Email: [lcanals@amgen.com](mailto:ctolman@amgen.com)

**Figures**

**Supplementary Figure 1.** Schematic diagram of study design

Abbreviations: PO, orally; QW, weekly; Q6M, every 6 months; SC, subcutaneously.

^a^ At least 1 prescription for denosumab or alendronate should be provided to the patient before signing the informed consent form. A patient needs to be enrolled in the study within 4 weeks after receiving the first denosumab injection or the first prescription of weekly alendronate for the current course of treatment.

**Supplementary Figure 2.** Study flow diagram

Singapore (n = 63)

- Denosumab, n = 42
- Alendronate, n = 21

Australia (n = 124)

- Denosumab, n = 59
- Alendronate, n = 65

Taiwan (n = 101)

- Denosumab, n = 56
- Alendronate, n = 45

Korea (n = 130)

- Denosumab, n = 71
- Alendronate, n = 59

Hong Kong (n = 131)

- Denosumab, n = 67
- Alendronate, n = 64

Drop-outs (n = 138)

- Denosumab, n = 56
- Alendronate, n = 82

Excluded (n = 18)

Completed 24-month visit (n = 549)

- Denosumab, n = 295
- Alendronate, n = 254

Enrolled (n = 687)

- Denosumab, n = 351
- Alendronate, n = 336

Screened (n = 705)

**Tables**

Supplementary Table 1: Subject accountability at 24-month visit.

|  | **Australia** | **Taiwan** | **South Korea** | **Hong Kong** | **Singapore** |
| --- | --- | --- | --- | --- | --- |
| **Number of sites, n** | 11 | 6 | 5 | 7 | 4 |
| **Number of enrolled subjects, n** | 157 | 152 | 154 | 155 | 69 |
| **Subject recruitment setting, n (%)** |  |  |  |  |  |
| Hospital | 60 (38.2%) | 152 (100%) | 154 (100%) | 85 (54.8%) | 69 (100%) |
| Clinic | 78 (49.7%) | 0 (0%) | 0 (0%) | 70 (45.2%) | 0 (0%) |
| Research center | 19 (12.1%) | 0 (0%) | 0 (0%) | 0 (0%) | 0 (0%) |
| Others | 0 (0%) | 0 (0%) | 0 (0%) | 0 (0%) | 0 (0%) |
| **Reasons for study exclusion, n (%)^a^** |  |  |  |  |  |
| Appropriate written informed consent was either not obtained or could not be provided due to disability | 0 (0%) | 0 (0%) | 0 (0%) | 0 (0%) | 0 (0%) |
| Not eligible for osteoporosis treatment based on local approved indication | 1 (0.6%) | 0 (0%) | 0 (0%) | 0 (0%) | 0 (0%) |
| Failure to enroll in study within 4 weeks after receiving first denosumab injection or weekly alendronate | 11 (6.5%) | 1 (0.6%) | 0 (0%) | 4 (2.5%) | 0 (0%) |
| Recent enrolment in another study involving investigational procedure, device, or drug | 0 (0%) | 0 (0%) | 0 (0%) | 0 (0%) | 0 (0%) |
| Contraindicated to both denosumab and alendronate treatment | 0 (0%) | 0 (0%) | 0 (0%) | 0 (0%) | 0 (0%) |
| History of noncompliance or did not complete previous course of osteoporosis treatment | 0 (0%) | 1 (0.6%) | 0 (0%) | 0 (0%) | 0 (0%) |
| **Dropouts, n (%)** |  |  |  |  |  |
| At 12 months | 14 (8.9%) | 43 (28.3%) | 30 (19.5%) | 14 (9.0%) | 5 (7.2%) |
| At 24 months | 33 (21.0%) | 51 (33.6%) | 24 (15.6%) | 24 (15.5%) | 6 (8.7%) |

^a^ Proportions are based on the total number of patients screened. The denominators for Australia, Taiwan, South Korea, Hong Kong, and Singapore are 169, 154, 154, 159, and 69, respectively.

**Supplementary Table 2.** Adjusted odds ratios and 95% confidence intervals for osteoporosis treatment persistence at 24 months (sensitivity analysis)

| **Territory** | **Adjusted odds ratio^a^** | **95% CI** | ***P*-value** | **Reference group^b^** |
| --- | --- | --- | --- | --- |
| **All territories, (N = 580)** |  |  |  |  |
| Denosumab group | 3.92 | (2.52, 6.10) | <0.001* | Alendronate |
| Age | 0.98 | (0.95, 1.00) | 0.047* | - |
| History of fracture | 0.67 | (0.44, 1.03) | 0.070 | no |
| Measurement of BMD | 1.26 | (0.78, 2.02) | 0.350 | no |
| Prior treatment for osteoporosis | 1.48 | (0.97, 2.27) | 0.069 | no |
| Prior treatment with oral glucocorticoids | 1.08 | (0.53, 2.17) | 0.841 | no |
| Reimbursement status^c^ | 0.62 | (0.37, 1.02) | 0.060 | No reimbursement |
| **Australia, (n = 142)** |  |  |  |  |
| Denosumab group | 9.25 | (3.72, 23.00) | <0.001* | Alendronate |
| Age | 0.99 | (0.94, 1.05) | 0.787 | - |
| History of fracture | 0.97 | (0.42, 2.29) | 0.953 | no |
| Measurement of BMD | 1.93 | (0.50, 7.54) | 0.343 | no |
| Prior treatment for osteoporosis | 1.38 | (0.53, 3.58) | 0.505 | no |
| Prior treatment with oral glucocorticoids | 0.82 | (0.23, 2.90) | 0.757 | no |
| **Taiwan, (n = 109)** |  |  |  |  |
| Denosumab group | 3.62 | (1.27, 10.34) | 0.016* | Alendronate |
| Age | 0.99 | (0.93, 1.05) | 0.655 | - |
| History of fracture | 0.38 | (0.11, 1.40) | 0.146 | no |
| Measurement of BMD | 1.71 | (0.47, 6.20) | 0.416 | no |
| Prior treatment for osteoporosis | 1.00 | (0.26, 3.79) | 0.997 | no |
| Prior treatment with oral glucocorticoids | 1.88 | (0.29, 12.00) | 0.505 | no |
| **South Korea, (n = 124)** |  |  |  |  |
| Denosumab group | 1.13 | (0.43, 2.99) | 0.805 | Alendronate |
| Age | 0.94 | (0.89, 1.00) | 0.035* | - |
| History of fracture | 0.39 | (0.16, 1.00) | 0.050* | no |
| Measurement of BMD | 1.79 | (0.56, 5.74) | 0.327 | no |
| Prior treatment for osteoporosis | 2.38 | (0.95, 6.01) | 0.065 | no |
| Prior treatment with oral glucocorticoids | 0.82 | (0.25, 2.67) | 0.741 | no |
| **Hong Kong, (n = 141)** |  |  |  |  |
| Denosumab group | 3.67 | (1.21, 11.12) | 0.021* | Alendronate |
| Age | 1.00 | (0.95, 1.06) | 0.961 | - |
| History of fracture | 1.11 | (0.42, 2.95) | 0.833 | no |
| Measurement of BMD | 2.96 | (0.78, 11.18) | 0.109 | no |
| Prior treatment for osteoporosis | 1.72 | (0.55, 5.40) | 0.350 | no |
| Prior treatment with oral glucocorticoids | 1.00 | (0.03, 28.83) | 0.998 | no |
| Reimbursement status^c^ | 1.25 | (0.36, 4.39) | 0.727 | No reimbursement |
| **Singapore, (n = 64)** |  |  |  |  |
| Denosumab group | 22.97 | (3.73, 141.57) | <0.001* | Alendronate |
| Age | 0.95 | (0.85, 1.06) | 0.359 | - |
| History of fracture | 0.85 | (0.19, 3.91) | 0.834 | no |
| Measurement of BMD | 2.06 | (0.37, 11.51) | 0.412 | no |
| Prior treatment for osteoporosis | 0.41 | (0.08, 2.06) | 0.280 | no |
| Prior treatment with oral glucocorticoids | 4.34 | (0.13, 151.08) | 0.417 | no |

^a^ Adjusted odds ratios were generated using multivariable logistic regression models. Treatment persistence was used as the dependent variable, while adjusting for treatment group, age, history of fracture, measurement of BMD, prior treatment for osteoporosis, and prior treatment with oral glucocorticoids.

^b^ The treatment group includes denosumab and alendronate. The history of fracture includes those with fracture and those without fracture. Measurement of BMD includes those with BMD measurements and those without. Prior treatment for osteoporosis includes individuals who were using and not using osteoporosis drugs before starting denosumab or alendronate treatment. Prior treatment with oral glucocorticoids includes individuals who were using and not using oral glucocorticoids before starting denosumab or alendronate treatment.

^c^ Further adjusted for baseline reimbursement status if there are at least 10% subjects in both treatment groups for reimbursement categories.

^*^ *P* < 0.05.

Abbreviations: BMD, bone mineral density; CI, confidence interval.

Supplementary Table 3. Osteoporosis treatment persistence at 24 months using multivariable logistic regression (sensitivity analysis using gap period of 30 days)

| **Territory** | **Adjusted odds ratio^a^** | **95% CI** | ***P*-value** | **Reference group^b^** |
| --- | --- | --- | --- | --- |
| **All territories (N = 580)** |  |  |  |  |
| Denosumab group | 5.27 | (3.45, 8.05) | <0.001* | Alendronate |
| Age | 0.97 | (0.95, 1.00) | 0.019* | - |
| History of fracture | 0.71 | (0.48, 1.06) | 0.091 | no |
| Measurement of BMD | 1.17 | (0.74, 1.86) | 0.506 | no |
| Prior treatment for osteoporosis | 1.59 | (1.06, 2.38) | 0.024* | no |
| Prior treatment with oral glucocorticoids | 0.95 | (0.49, 1.83) | 0.867 | no |
| **Australia (n = 142)** |  |  |  |  |
| Denosumab group | 17.46 | (6.68, 45.64) | <0.001* | Alendronate |
| Age | 1.00 | (0.94, 1.06) | 0.927 | - |
| History of fracture | 1.08 | (0.43, 2.71) | 0.877 | no |
| Measurement of BMD | 1.85 | (0.45, 7.62) | 0.397 | no |
| Prior treatment for osteoporosis | 1.14 | (0.41, 3.16) | 0.796 | no |
| Prior treatment with oral glucocorticoids | 0.65 | (0.18, 2.40) | 0.520 | no |
| **Taiwan (n = 109)** |  |  |  |  |
| Denosumab group | 5.63 | (2.03, 15.63) | <0.001* | Alendronate |
| Age | 0.97 | (0.91, 1.03) | 0.314 | - |
| History of fracture | 0.53 | (0.16, 1.72) | 0.287 | no |
| Measurement of BMD | 1.58 | (0.46, 5.37) | 0.465 | no |
| Prior treatment for osteoporosis | 1.31 | (0.37, 4.60) | 0.676 | no |
| Prior treatment with oral glucocorticoids | 1.13 | (0.23, 5.55) | 0.881 | no |
| **South Korea (n = 124)** |  |  |  |  |
| Denosumab group | 1.23 | (0.50, 3.02) | 0.652 | Alendronate |
| Age | 0.96 | (0.91, 1.01) | 0.126 | - |
| History of fracture | 0.49 | (0.20, 1.18) | 0.113 | no |
| Measurement of BMD | 2.17 | (0.74, 6.41) | 0.161 | no |
| Prior treatment for osteoporosis | 2.31 | (0.98, 5.41) | 0.055 | no |
| Prior treatment with oral glucocorticoids | 1.16 | (0.37, 3.60) | 0.802 | no |
| **Hong Kong (n = 141)** |  |  |  |  |
| Denosumab group | 3.74 | (1.34, 10.39) | 0.012* | Alendronate |
| Age | 0.98 | (0.94, 1.04) | 0.541 | - |
| History of fracture | 1.43 | (0.57, 3.57) | 0.448 | no |
| Measurement of BMD | 1.49 | (0.54, 4.11) | 0.442 | no |
| Prior treatment for osteoporosis | 1.95 | (0.67, 5.66) | 0.219 | no |
| Prior treatment with oral glucocorticoids | 1.58 | (0.07, 37.26) | 0.778 | no |
| **Singapore (n = 64)** |  |  |  |  |
| Denosumab group | 47.06 | (6.61, 335.08) | <0.001* | Alendronate |
| Age | 0.94 | (0.84, 1.05) | 0.251 | - |
| History of fracture | 0.93 | (0.19, 4.52) | 0.925 | no |
| Measurement of BMD | 2.60 | (0.41, 16.63) | 0.312 | no |
| Prior treatment for osteoporosis | 0.56 | (0.11, 2.84) | 0.485 | no |
| Prior treatment with oral glucocorticoids | 10.03 | (0.25, 403.55) | 0.221 | no |

^a^ Adjusted odds ratios were generated using multivariable logistic regression models. Treatment persistence was used as the dependent variable, while adjusting for treatment group, age, history of fracture, measurement of BMD, prior treatment for osteoporosis, and prior treatment with oral glucocorticoids.

^b^ The treatment group includes denosumab and alendronate. The history of fracture includes those with fractures and those without fractures. Measurement of BMD includes those with BMD measurements and those without them. Prior treatment for osteoporosis includes individuals who were using and not using osteoporosis drugs before starting denosumab or alendronate treatment. Prior treatment with oral glucocorticoids includes individuals who were using or not using oral glucocorticoids before starting denosumab or alendronate treatment.

^*^ *P* < 0.05.

Abbreviations: BMD, bone mineral density; CI, confidence interval.

Supplementary Table 4. Osteoporosis treatment persistence at 24 months using multivariable logistic regression (sensitivity analysis using gap period of 90 days)

| **Territory** | **Adjusted odds ratio^a^** | **95% CI** | ***P*-value** | **Reference group^b^** |
| --- | --- | --- | --- | --- |
| **All territories (N = 580)** |  |  |  |  |
| Denosumab group | 2.85 | (1.82, 4.47) | <0.001* | Alendronate |
| Age | 0.98 | (0.95, 1.00) | 0.046* | - |
| History of fracture | 0.60 | (0.39, 0.92) | 0.018* | no |
| Measurement of BMD | 1.08 | (0.66, 1.76) | 0.761 | no |
| Prior treatment for osteoporosis | 1.45 | (0.94, 2.25) | 0.094 | no |
| Prior treatment with oral glucocorticoids | 0.96 | (0.47, 1.95) | 0.904 | no |
| **Australia (n = 142)** |  |  |  |  |
| Denosumab group | 10.47 | (4.03, 27.18) | <0.001* | Alendronate |
| Age | 0.98 | (0.93, 1.04) | 0.533 | - |
| History of fracture | 0.81 | (0.34, 1.95) | 0.644 | no |
| Measurement of BMD | 2.29 | (0.55, 9.45) | 0.254 | no |
| Prior treatment for osteoporosis | 1.89 | (0.71, 5.03) | 0.205 | no |
| Prior treatment with oral glucocorticoids | 0.69 | (0.19, 2.52) | 0.576 | no |
| **Taiwan (n = 109)** |  |  |  |  |
| Denosumab group | 1.12 | (0.34, 3.70) | 0.856 | Alendronate |
| Age | 0.97 | (0.90, 1.04) | 0.342 | - |
| History of fracture | 0.10 | (0.01, 1.30) | 0.078 | no |
| Measurement of BMD | 0.90 | (0.15, 5.43) | 0.907 | no |
| Prior treatment for osteoporosis | 0.54 | (0.09, 3.42) | 0.515 | no |
| Prior treatment with oral glucocorticoids | 5.26 | (0.29, 94.16) | 0.259 | no |
| **South Korea (n = 124)** |  |  |  |  |
| Denosumab group | 0.88 | (0.32, 2.38) | 0.800 | Alendronate |
| Age | 0.95 | (0.89, 1.00) | 0.050 | - |
| History of fracture | 0.39 | (0.15, 1.02) | 0.055 | no |
| Measurement of BMD | 1.99 | (0.61, 6.42) | 0.252 | no |
| Prior treatment for osteoporosis | 2.23 | (0.87, 5.72) | 0.094 | no |
| Prior treatment with oral glucocorticoids | 0.79 | (0.24, 2.58) | 0.691 | no |
| **Hong Kong (n = 141)** |  |  |  |  |
| Denosumab group | 3.04 | (0.99, 9.38) | 0.053 | Alendronate |
| Age | 1.01 | (0.96, 1.07) | 0.701 | - |
| History of fracture | 1.43 | (0.51, 4.02) | 0.497 | no |
| Measurement of BMD | 2.93 | (0.96, 8.89) | 0.058 | no |
| Prior treatment for osteoporosis | 1.38 | (0.43, 4.43) | 0.587 | no |
| Prior treatment with oral glucocorticoids | 1.50 | (0.06, 36.43) | 0.804 | no |
| **Singapore (n = 64)** |  |  |  |  |
| Denosumab group | 12.22 | (2.15, 69.29) | 0.005* | Alendronate |
| Age | 0.96 | (0.86, 1.07) | 0.414 | - |
| History of fracture | 0.51 | (0.11, 2.35) | 0.391 | no |
| Measurement of BMD | 1.28 | (0.23, 7.05) | 0.780 | no |
| Prior treatment for osteoporosis | 0.43 | (0.09, 2.17) | 0.310 | no |
| Prior treatment with oral glucocorticoids | 3.22 | (0.09, 114.26) | 0.521 | no |

^a^ Adjusted odds ratios were generated using multivariable logistic regression models. Treatment persistence was used as the dependent variable, while adjusting for treatment group, age, history of fracture, measurement of BMD, prior treatment for osteoporosis, and prior treatment with oral glucocorticoids.

^b^ The treatment group includes denosumab and alendronate. The history of fracture includes those with fractures and those without fractures. Measurement of BMD includes those with BMD measurements and those without fracture. Prior treatment for osteoporosis includes individuals who were using and not using osteoporosis drugs before starting denosumab or alendronate treatment. Prior treatment with oral glucocorticoids includes individuals who were using or not using oral glucocorticoids before starting denosumab or alendronate treatment.

^*^ *P* < 0.05.

Abbreviations: BMD, bone mineral density; CI, confidence interval.

**Supplementary Table 5.** Associations between variables and changes in T-score from baseline using multivariable linear regression at 24 months

| **Variable** | **Regression Coefficient (95% CI)** | | |
| --- | --- | --- | --- |
|  | **Lumbar Spine** | **Total Hip** | **Femoral Neck** |
| Treatment | | | |
| Denosumab | -0.17 (-0.37, 0.04) | -0.03 (-0.09, 0.04) | 0.02 (-0.09, 0.13) |
| Alendronate | Reference | Reference | Reference |
| Age | 0.01 (0.00, 0.02) | 0.00 (-0.01, 0.00) | 0.00 (-0.01, 0.00) |
| History of fracture | | | |
| Yes | 0.23 (0.00, 0.46)* | -0.03 (-0.10, 0.05) | -0.05 (-0.17, 0.07) |
| No | Reference | Reference | Reference |
| Baseline BMD | -0.15 (-0.25, -0.04)* | -0.05 (-0.10, -0.01)* | -0.10 (-0.18, -0.03)* |
| Prior treatment for osteoporosis | | | |
| Yes | -0.39 (-0.62, -0.15)* | -0.07 (-0.15, 0.00) | -0.12 (-0.24, 0.00) |
| No | Reference | Reference | Reference |
| Prior treatment with oral glucocorticoids | | | |
| Yes | 0.04 (-0.26, 0.33) | 0.04 (-0.06, 0.14) | 0.09 (-0.07, 0.26) |
| No | Reference | Reference | Reference |
| Model of measuring device | | | |
| GE-Lunar | 0.32 (-0.09, 0.73) | 0.13 (-0.02, 0.27) | 0.11 (-0.12, 0.33) |
| Hologic | 0.25 (-0.19, 0.69) | 0.08 (-0.08, 0.23) | 0.07 (-0.18, 0.31) |
| Missing | Reference | Reference | Reference |
| History of falls | | | |
| Yes | -0.11 (-0.39, 0.18) | 0.01 (-0.08, 0.11) | -0.01 (-0.16, 0.13) |
| No | Reference | Reference | Reference |
| Body Mass Index | -0.01 (-0.04, 0.01) | 0.00 (-0.01, 0.01) | 0.01 (-0.01, 0.02) |
| Smoking status | | | |
| Current smoker | 0.45 (-0.18, 1.08) | 0.10 (-0.13, 0.33) | -0.06 (-0.40, 0.29) |
| Former smoker | -0.05 (-0.49, 0.39) | 0.03 (-0.12, 0.18) | 0.10 (-0.14, 0.35) |
| Never | Reference | Reference | Reference |
| Ethnicity | | | |
| Caucasian | 0.16 (-0.19, 0.51) | -0.02 (-0.13, 0.10) | -0.06 (-0.24, 0.13) |
| Korean | 0.05 (-0.19, 0.30) | -0.06 (-0.13, 0.02) | -0.05 (-0.18, 0.07) |
| Others^a^ | 0.12 (-0.49, 0.74) | 0.01 (-0.20, 0.22) | -0.10 (-0.44, 0.23) |
| Chinese | Reference | Reference | Reference |

^a^ Malays and Indians were included in others.

^*^ *P* < 0.05.

Abbreviations: BMD, bone mineral density; CI, confidence interval.

**Supplementary Table 6.** Associations between variables and percentage changes in T-scores from baseline using multivariable linear regression at 24 months

| **Variable** | **Regression coefficient (95% CI)** | | |
| --- | --- | --- | --- |
|  | **Lumbar spine** | **Total hip** | **Femoral neck** |
| Treatment | | | |
| Denosumab | -5.67 (-17.69, 6.35) | -0.99 (-5.93, 3.96) | 6.01 (-1.04, 13.06) |
| Alendronate | Reference | Reference | Reference |
| Age | 0.65 (-0.07, 1.36) | -0.25 (-0.55, 0.04) | -0.23 (-0.64, 0.19) |
| History of fracture | | | |
| Yes | 7.81 (-5.51, 21.14) | -1.49 (-6.89, 3.91) | -3.93 (-11.68, 3.81) |
| No | Reference | Reference | Reference |
| Baseline BMD | 12.24 (6.28, 18.19)* | 0.93 (-2.48, 4.34) | -3.27 (-8.31, 1.77) |
| Prior treatment for osteoporosis | | | |
| Yes | -22.33 (-35.88, -8.78)* | -1.80 (-7.34, 3.74) | -6.01 (-13.94, 1.91) |
| No | Reference | Reference | Reference |
| Prior treatment with oral glucocorticoids | | | |
| Yes | 17.81 (0.79, 34.82)* | 1.01 (-6.45, 8.47) | 6.46 (-4.17, 17.08) |
| No | Reference | Reference | Reference |
| Model of measuring device | | | |
| GE-Lunar | 32.88 (9.17, 56.59)* | 7.74 (-2.83, 18.32) | 11.17 (-3.28, 25.61) |
| Hologic | 26.28 (0.92, 51.64)* | 1.55 (-9.99, 13.08) | 8.25 (-7.62, 24.13) |
| Missing | Reference | Reference | Reference |
| History of falls | | | |
| Yes | 0.47 (-15.79, 16.72) | -0.38 (-7.21, 6.44) | -1.79 (-11.40, 7.82) |
| No | Reference | Reference | Reference |
| Body Mass Index | -1.57 (-3.09, -0.05)* | 0.30 (-0.36, 0.95) | 0.29 (-0.63, 1.20) |
| Smoking status | | | |
| Current smoker | 13.59 (-22.93, 50.11) | 8.73 (-8.37, 25.84) | -4.55 (-27.18, 18.07) |
| Former smoker | -29.43 (-54.94, -3.92)* | 4.41 (-6.48, 15.30) | -1.28 (-17.05, 14.49) |
| Never | Reference | Reference | Reference |
| Ethnicity | | | |
| Caucasian | 28.23 (7.76, 48.70)* | -1.48 (-10.15, 7.18) | 2.09 (-10.11, 14.28) |
| Korean | 11.35 (-2.67, 25.38) | -4.09 (-9.65, 1.46) | -6.18 (-14.31, 1.95) |
| Others^a^ | 24.65 (-10.66, 59.96) | 1.88 (-14.00, 17.75) | -14.58 (-36.42, 7.25) |
| Chinese | Reference | Reference | Reference |

^a^ Malays and Indians were included in others.

^*^ *P* < 0.05.

Abbreviations: BMD, bone mineral density; CI, confidence interval.
